# Supplementary material for: Sequence variation at ANAPC1 accounts for 24% of the variability in corneal endothelial cell density
Source: Nat Commun. 2019 Mar 20;10:1284. doi: 10.1038/s41467-019-09304-9 (PMC6427039; doi:10.1038/s41467-019-09304-9)
Supplement: Supplementary file 1 — Supplementary Information [file 41467_2019_9304_MOESM1_ESM.pdf]

Supplementary Information

Sequence variation at *ANAPC1* accounts for 24% of the variability in corneal endothelial cell density

Ivarsdottir et al.

Supplementary Figures

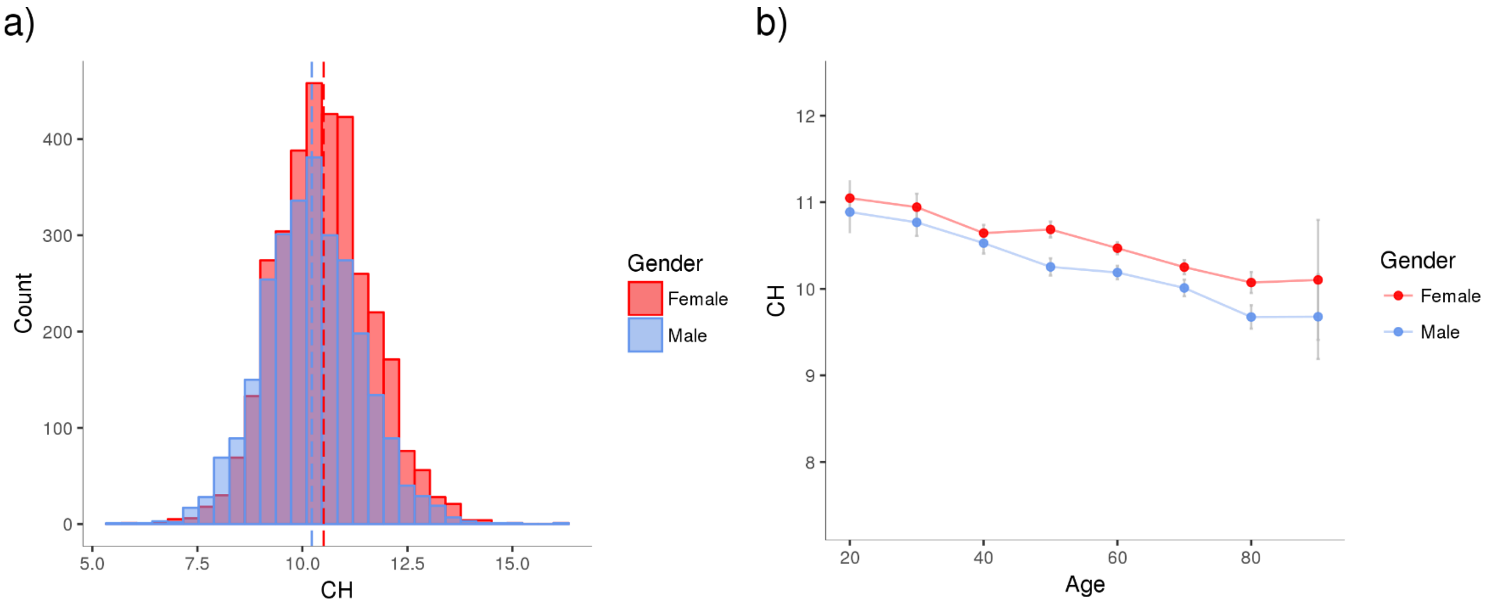

Supplementary Figure 1. Corneal hysteresis (CH) by age and sex (N=6,125). a) Histograms showing the distribution of CH measurements for each sex. **b)** The average CH values for subjects belonging to a 10 year age group (e.g. Age=30 for individuals between 26 and 35) against age, for men and women. The grey lines show the 95% confidence intervals.

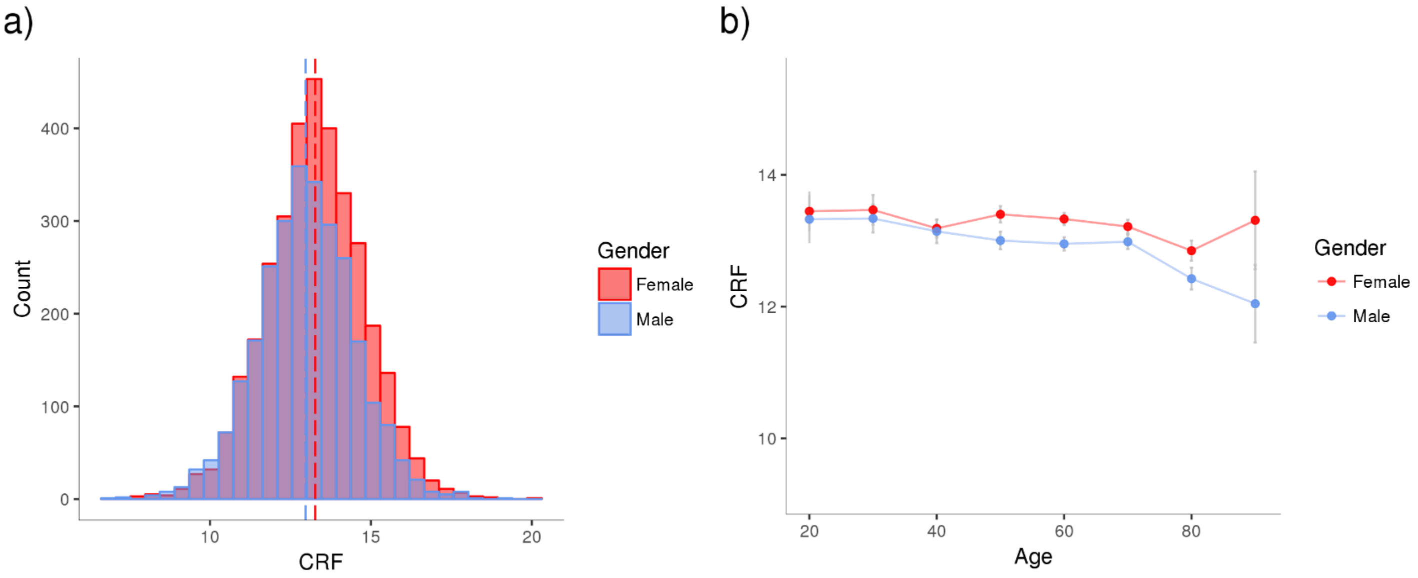

Supplementary Figure 2. Corneal resistance factor (CRF) by age and sex (N=6,125). a) Histograms showing the distribution of CRF measurements for each sex. **b)** The average CRF values for subjects belonging to a 10 year age group (e.g. Age=30 for individuals between 26 and 35) against age, for men and women. The grey lines show the 95% confidence intervals.

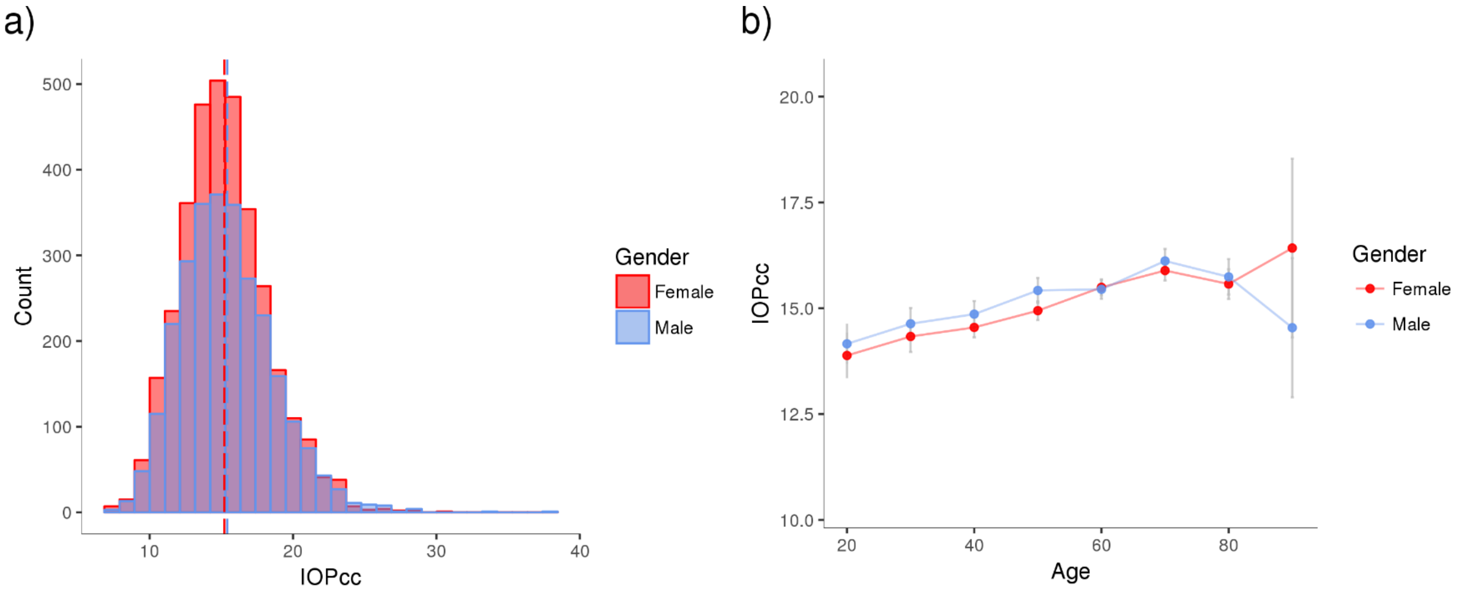

Supplementary Figure 3. Corneal compensated intraocular pressure (IOPcc) by age and sex (N=6,125). a) Histograms showing the distribution of IOPcc measurements for each sex. **b)** The average IOPcc values for subjects belonging to a 10 year age group (e.g. Age=30 for individuals between 26 and 35) against age, for men and women. The grey lines show the 95% confidence intervals.

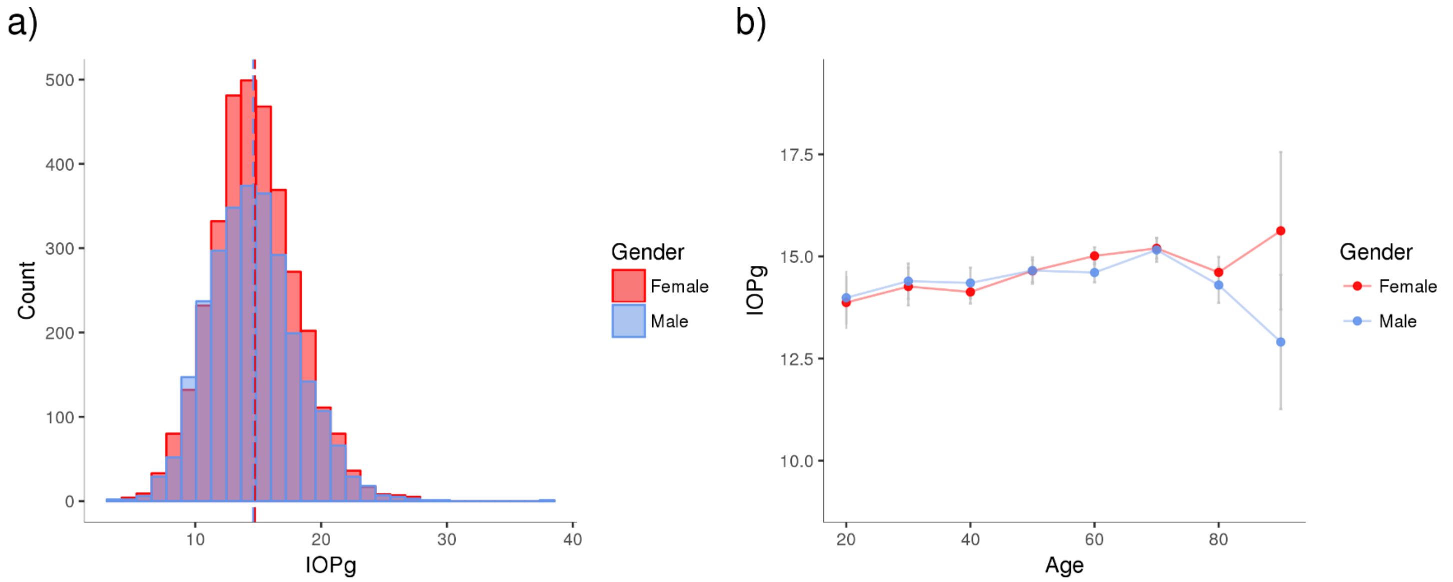

Supplementary Figure 4. Goldmann correlated intraocular pressure (IOPg) by age and sex (N=6,125). a) Histograms showing the distribution of IOPg measurements for each sex. **b)** The average IOPg values for subjects belonging to a 10 year age group (e.g. Age=30 for individuals between 26 and 35) against age, for men and women. The grey lines show the 95% confidence intervals.

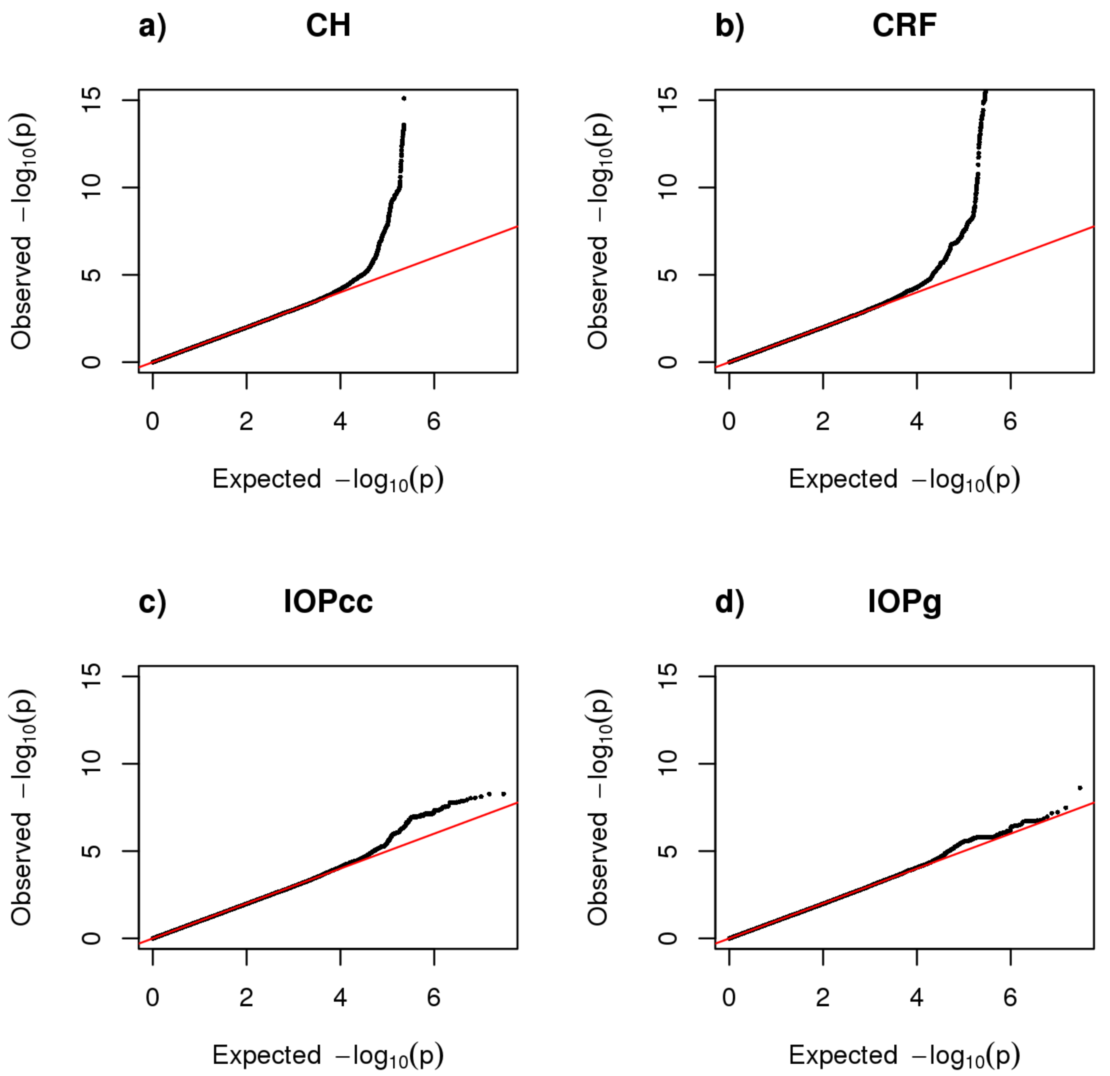

Supplementary Figure 5. Q-Q plot for GWAS results for ocular biomechanics; CH, CRF, IOPcc and IOPg. The y-axis shows the observed $–\log_{10}P$ values after adjustment using LD score regression. The observed values were rankled and for each rank the expected $–\log_{10}P$ value was determined. The correction factors are a) 1.06, b) 1.05, c) 1.05 and d) 1.05.

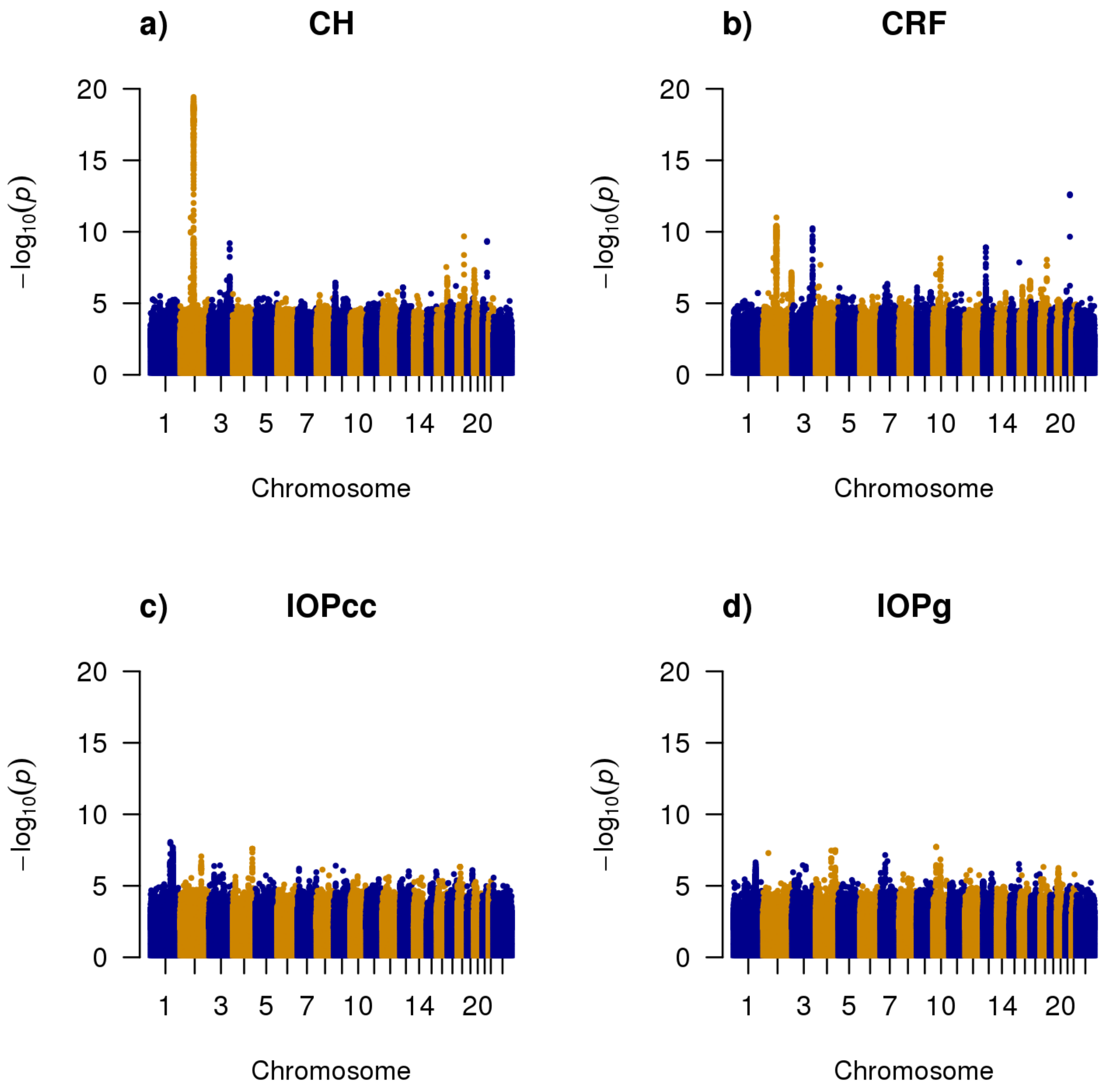

Supplementary Figure 6. Manhattan plots for the GWAS (N=6,125) on ocular biomechanics obtained from the ocular response analyzer. **a)** CH, **b)** CRF, **c)** IOPcc and **d)** IOPg. The -$\log_{10}$ *P*-values are plotted for each variant against their chromosomal position.

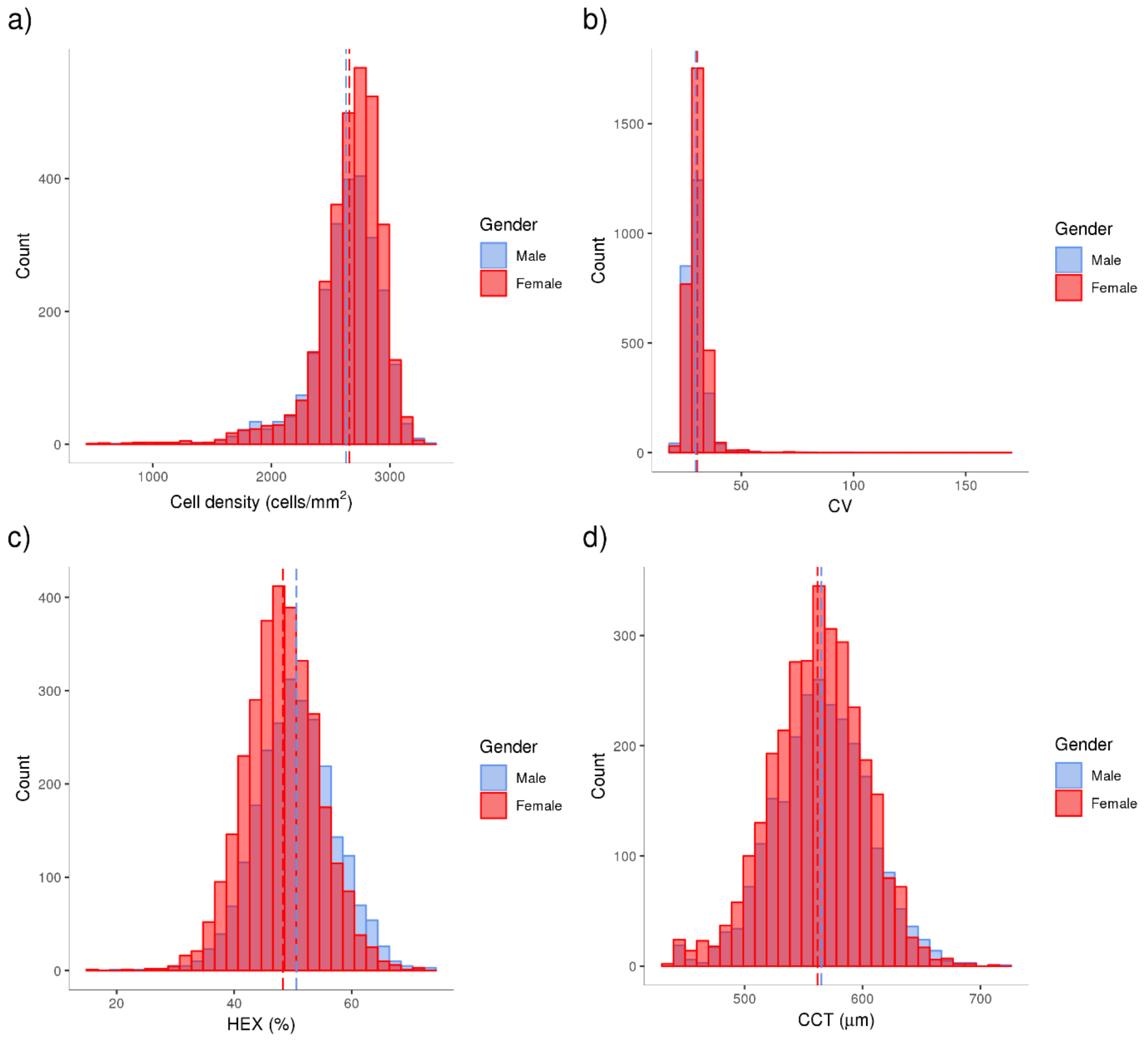

Supplementary Figure 7. Distribution plots for structural corneal measures. Histograms showing the distribution of **a)** cell density, **b)** coefficient of cell size variation (CV), **c)** percentage of hexagonal cells (HEX) and **d)** central corneal thickness (CCT) measurements for each sex.

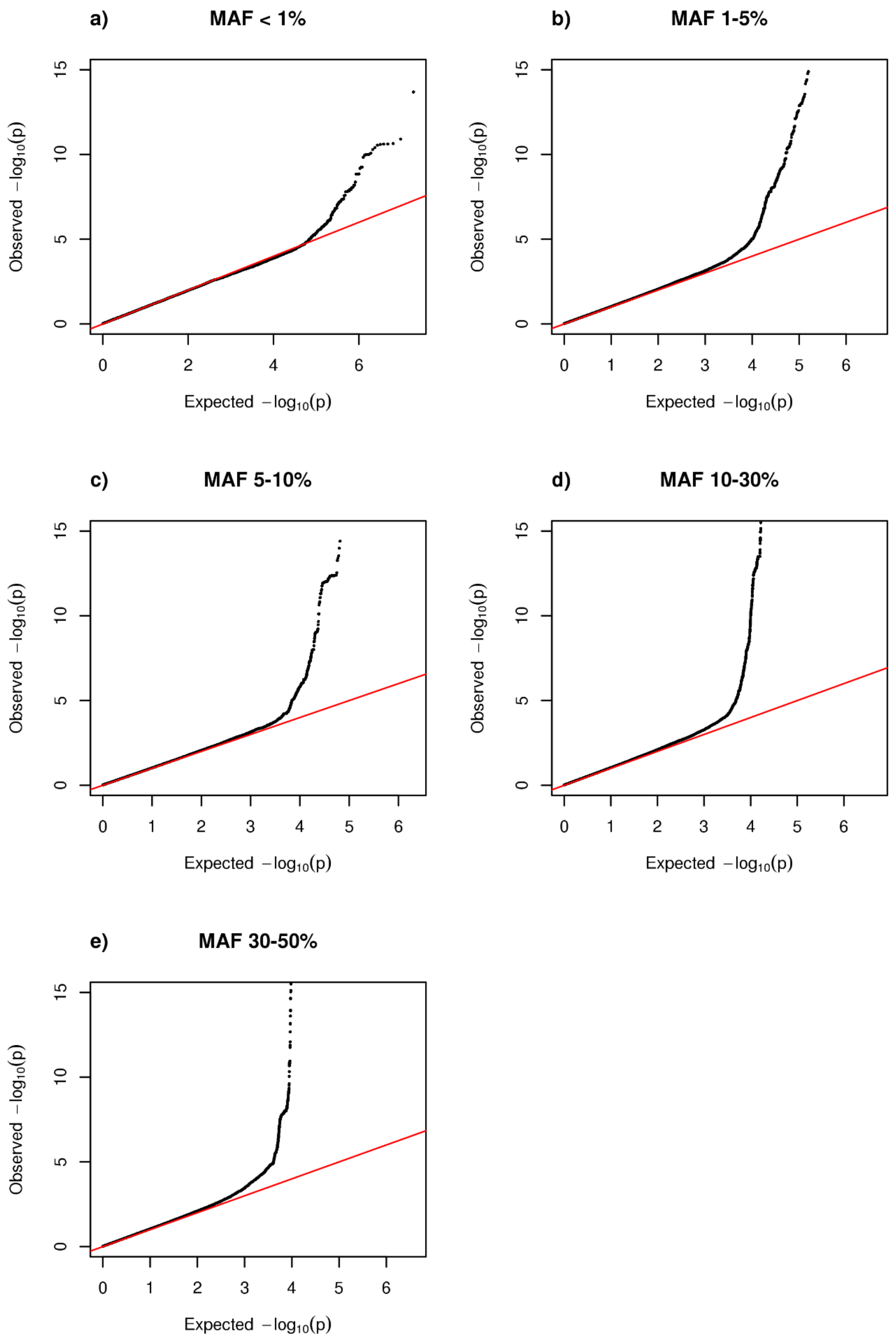

Supplementary Figure 8. Q-Q plot for GWAS results for cell density stratified by allele frequency. The y-axis shows the observed $-\log_{10}P$ values after adjustment using LD score regression (correction factor = 1.05). The observed values were rankled and for each rank the expected $-\log_{10}P$ value was determined.

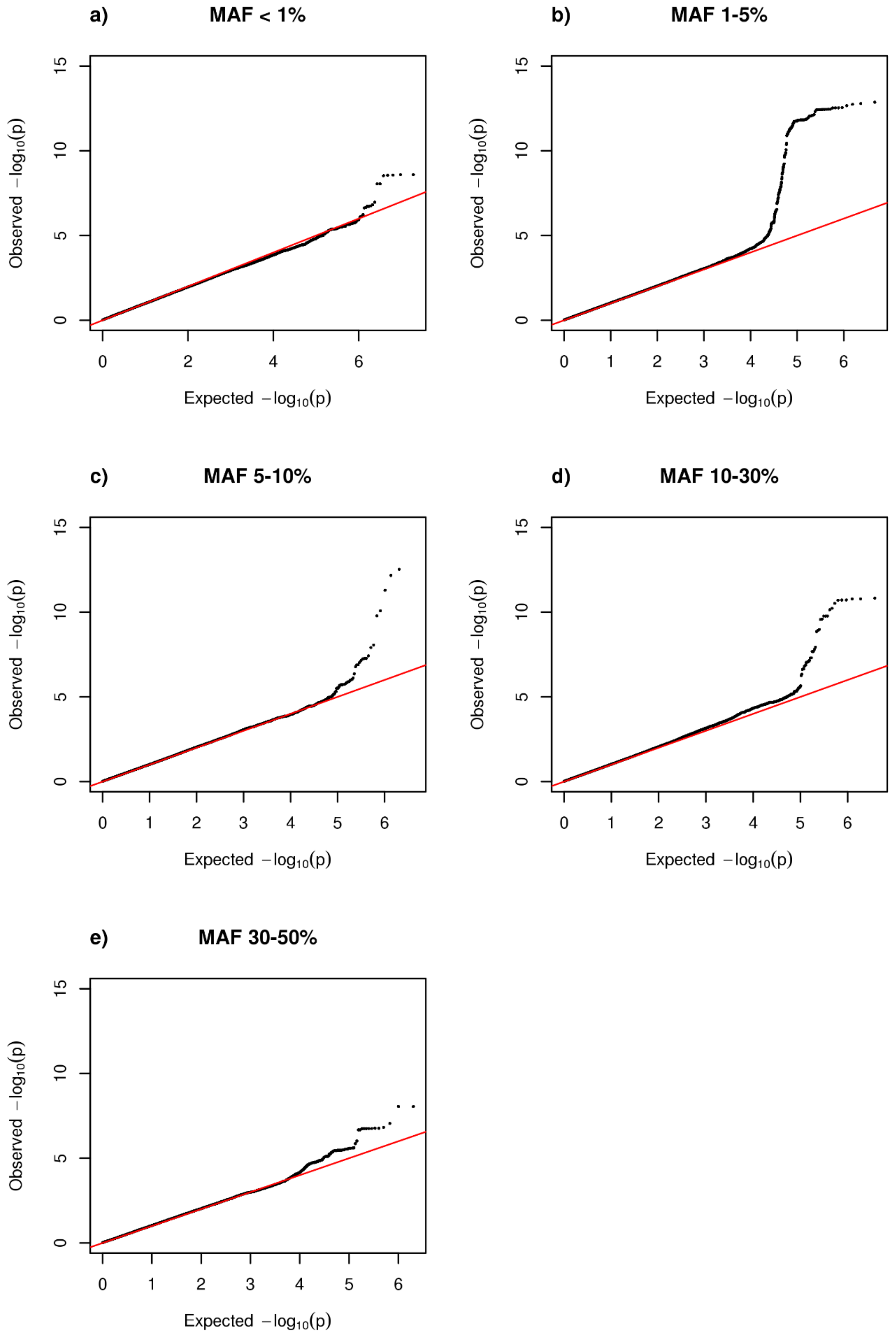

Supplementary Figure 9. Q-Q plot for GWAS results for HEX stratified by allele frequency. The y-axis shows the observed $–\log_{10}P$ values after adjustment using LD score regression (correction factor = 1.03). The observed values were rankled and for each rank the expected $–\log_{10}P$ value was determined.

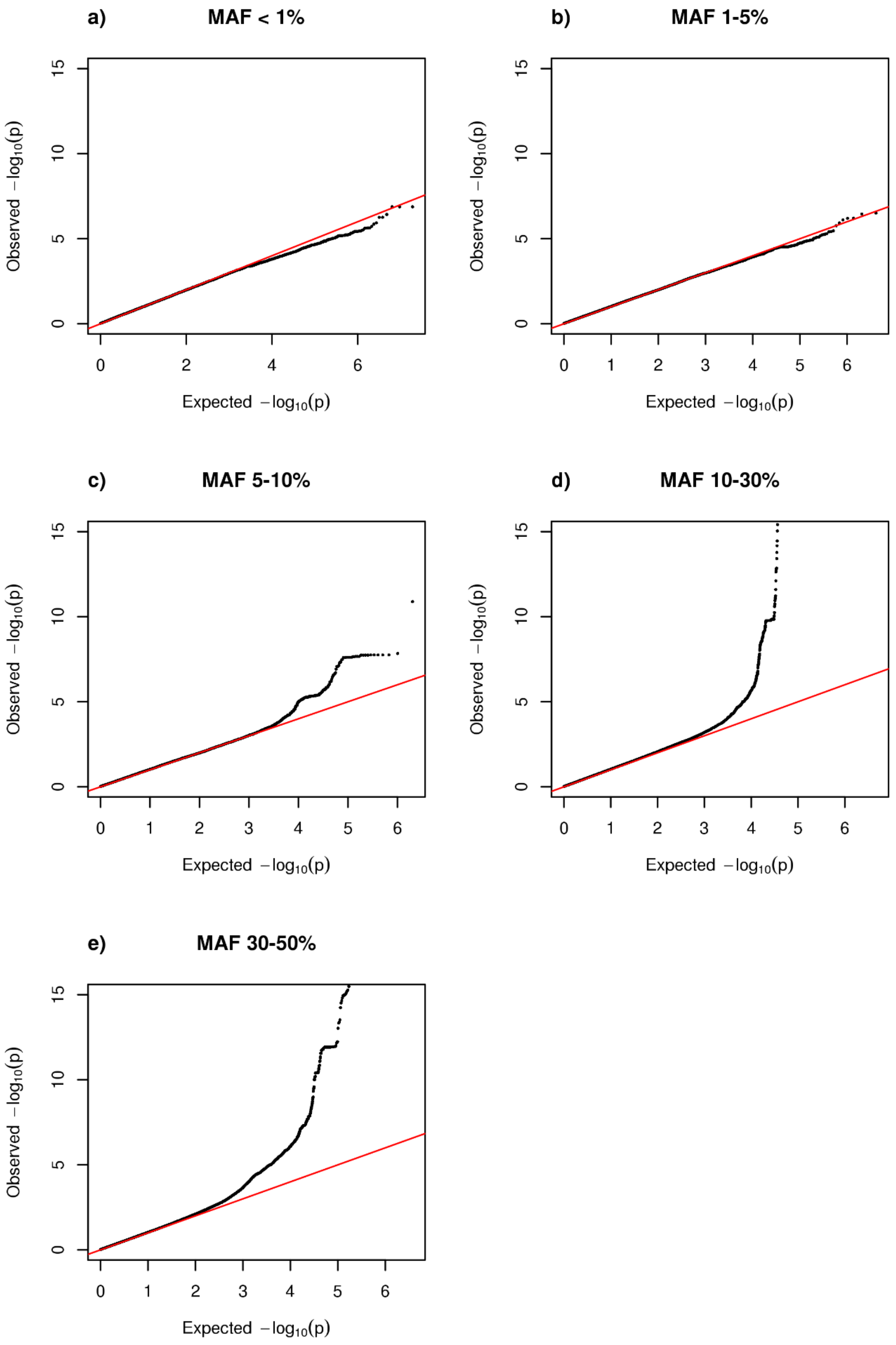

Supplementary Figure 10. Q-Q plot for GWAS results for CV stratified by allele frequency. The y-axis shows the observed $–\log_{10}P$ values after adjustment using LD score regression (correction factor = 1.03). The observed values were rankled and for each rank the expected $–\log_{10}P$ value was determined.

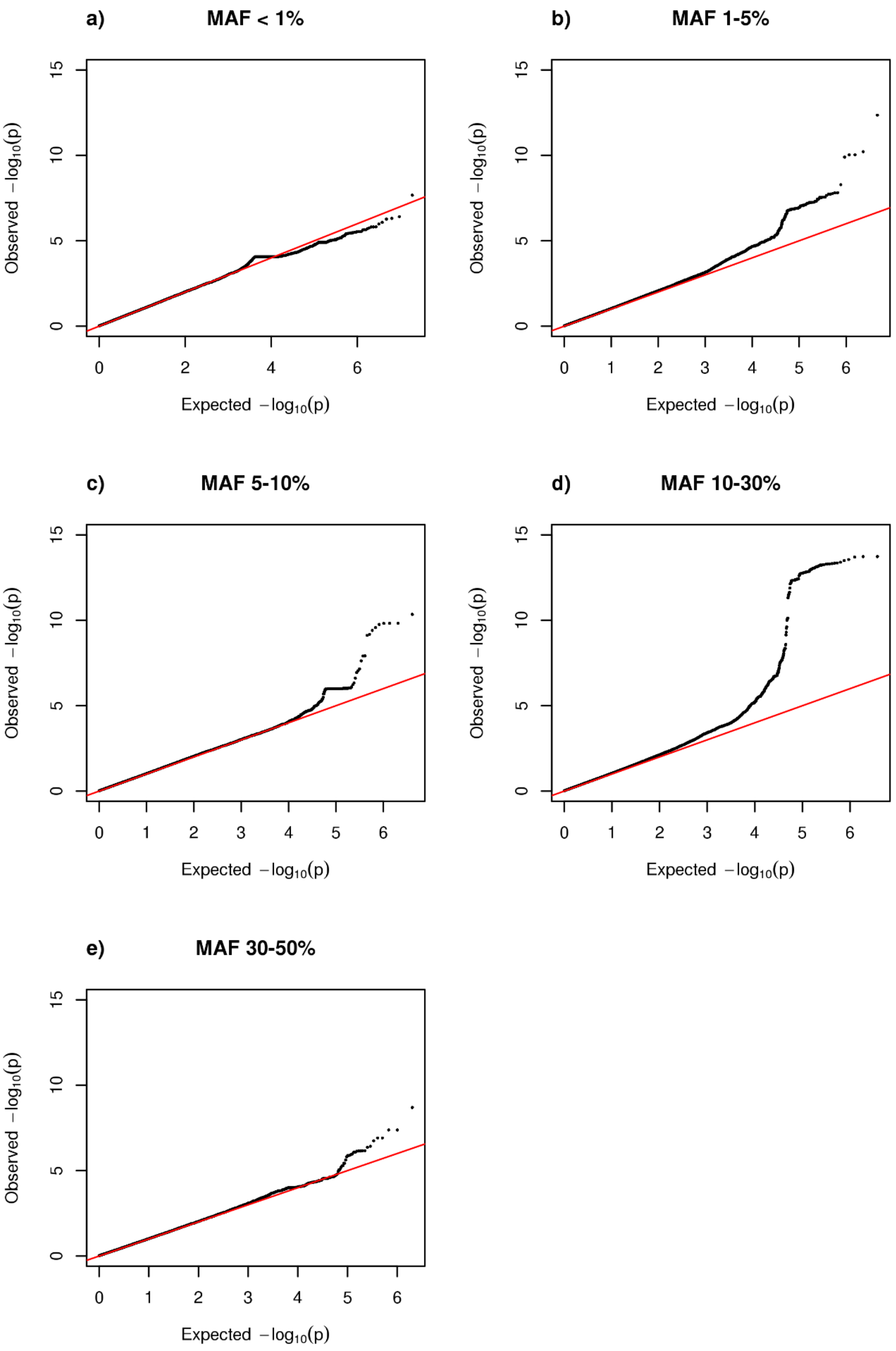

Supplementary Figure 11. Q-Q plot for GWAS results for CCT stratified by allele frequency. The y-axis shows the observed $–\log_{10}P$ values after adjustment using LD score regression (correction factor = 1.06). The observed values were rankled and for each rank the expected $–\log_{10}P$ value was determined.

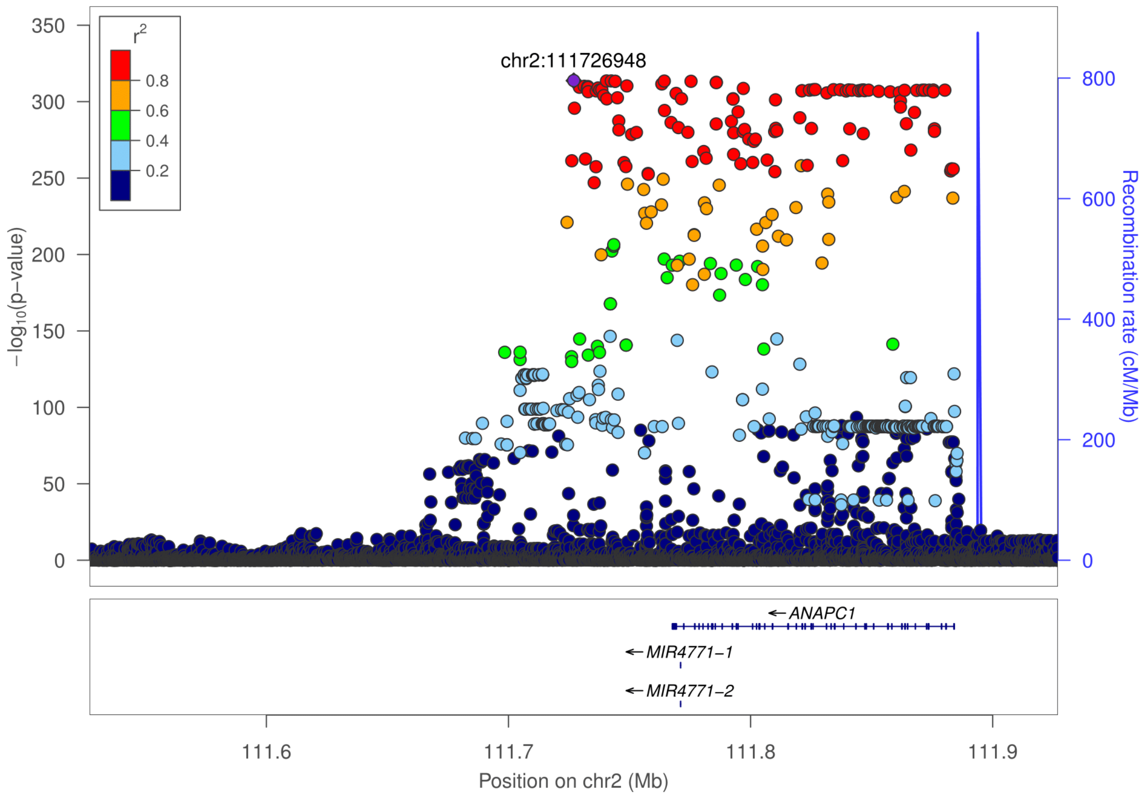

Supplementary Figure 12. Locus-plot showing variants at the *ANAPC1* locus (hg38) associating with cell density. The leading variant, rs78658973[A], is labelled as a purple diamond, other variants are colored according to correlation (r^2) with the leading marker. $-\log_{10}$ *P*-values are shown on the left y-axis and correspond to the variants depicted in the plot. The right y-axis shows calculated recombination rates at the chromosomal location, plotted as a blue line.

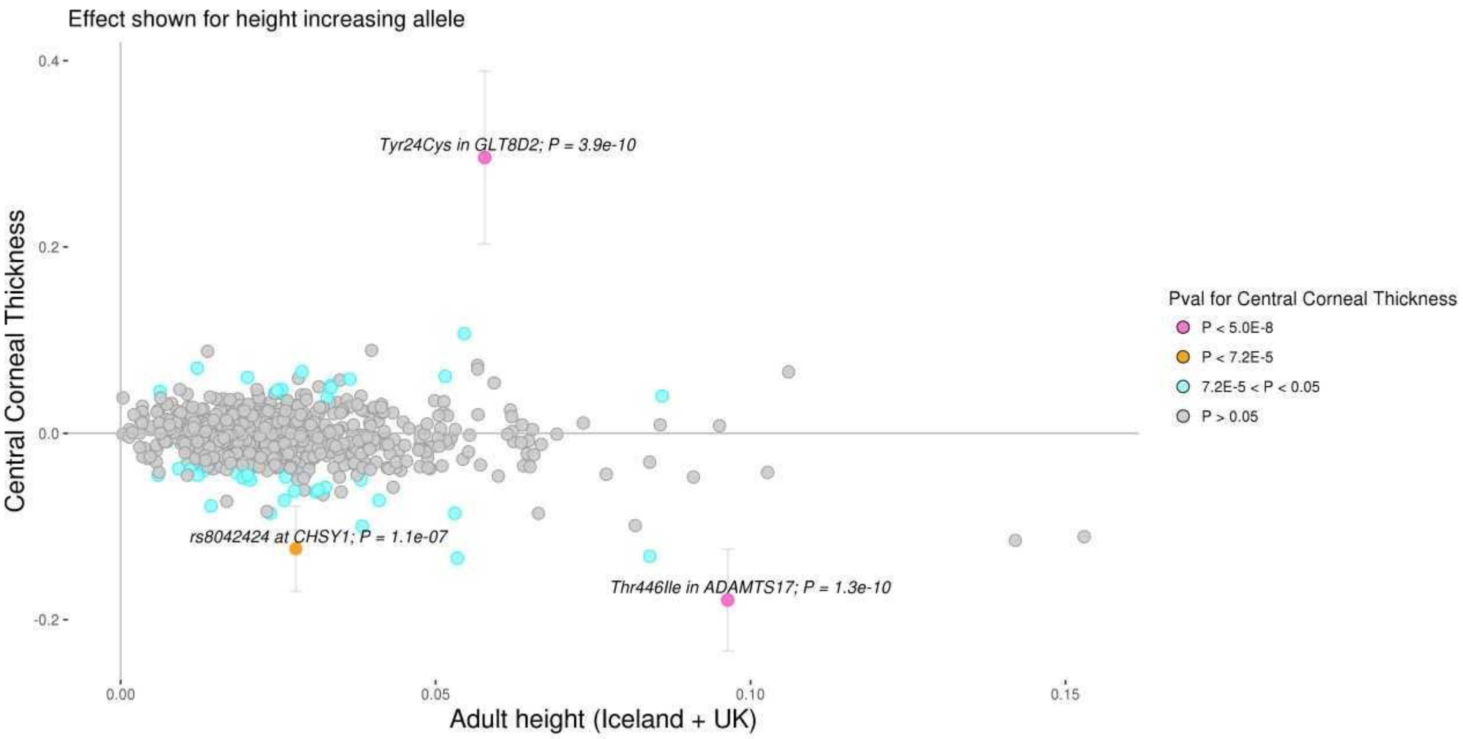

Supplementary Figure 13. The effect on height vs. effect on central corneal thickness (CCT) for known height variants.

Scatter plot showing the effect of 693 variants reported by GIANT[1], as well as the two novel CCT variants in *GLT8D2* and *ADAMTS17*, on adult height against their effect on CCT in our data. The effects are shown for the allele that is associates with increased height in our data. Different colors of points represent their *P*-values for their association with CCT. The grey lines show the 95% confidence intervals.

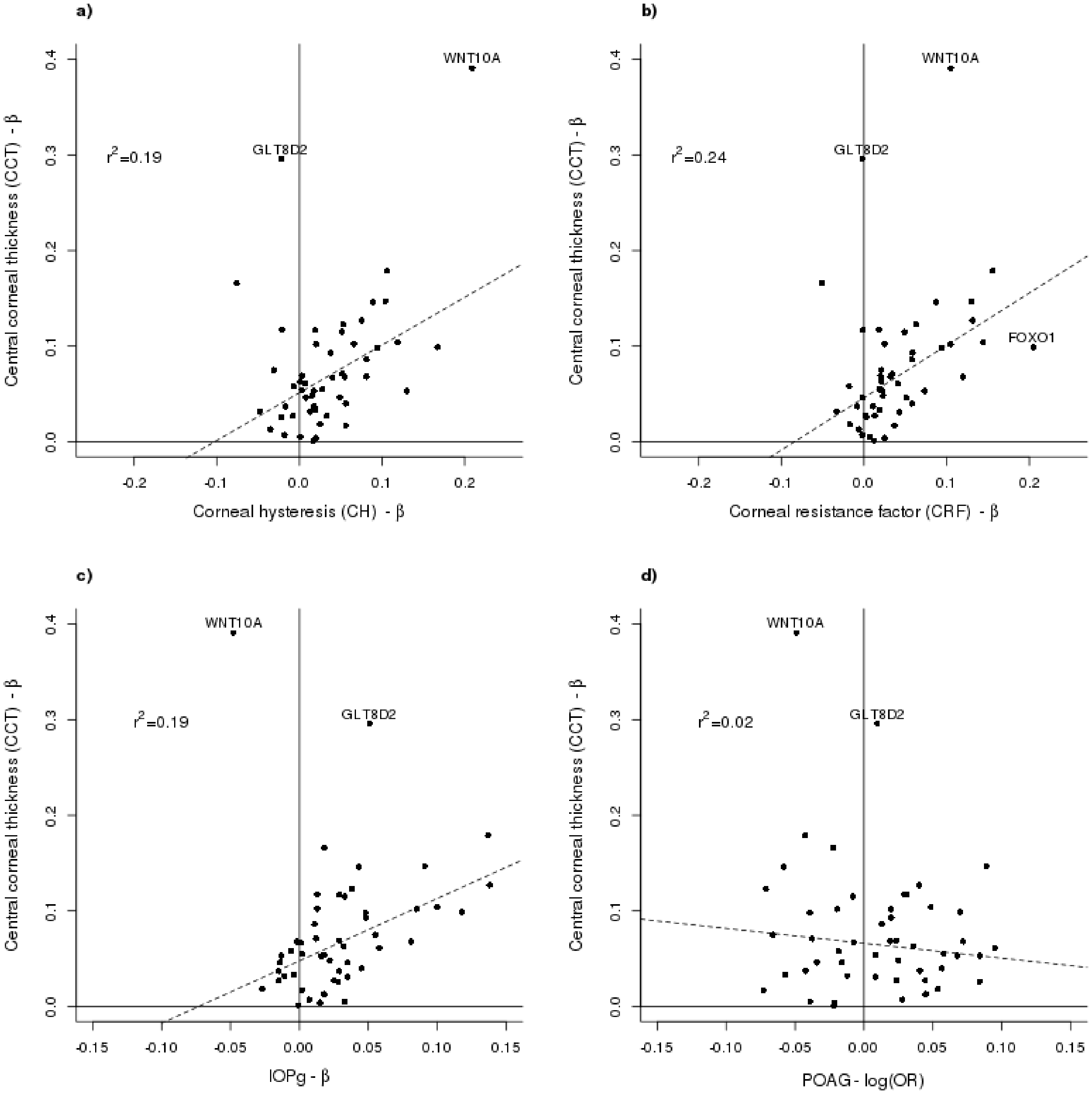

Supplementary Figure 14. Reported central corneal thickness (CCT) variants and their effect on CCT vs their effect on corneal hysteresis (CH), corneal resistance factor (CRF), Goldmann correlated intraocular pressure (IOPg) and primary open angle glaucoma (POAG). Scatter plot showing the effect of 48 variants reported to associate with CCT[2,3], as well as the two novel CCT variants in *GLT8D2* and *ADAMTS17*, on CCT against their effect on a) CH, b) CRF, c) IOPg and d) POAG in our data. Effects are given for the allele that increases CCT in our data.

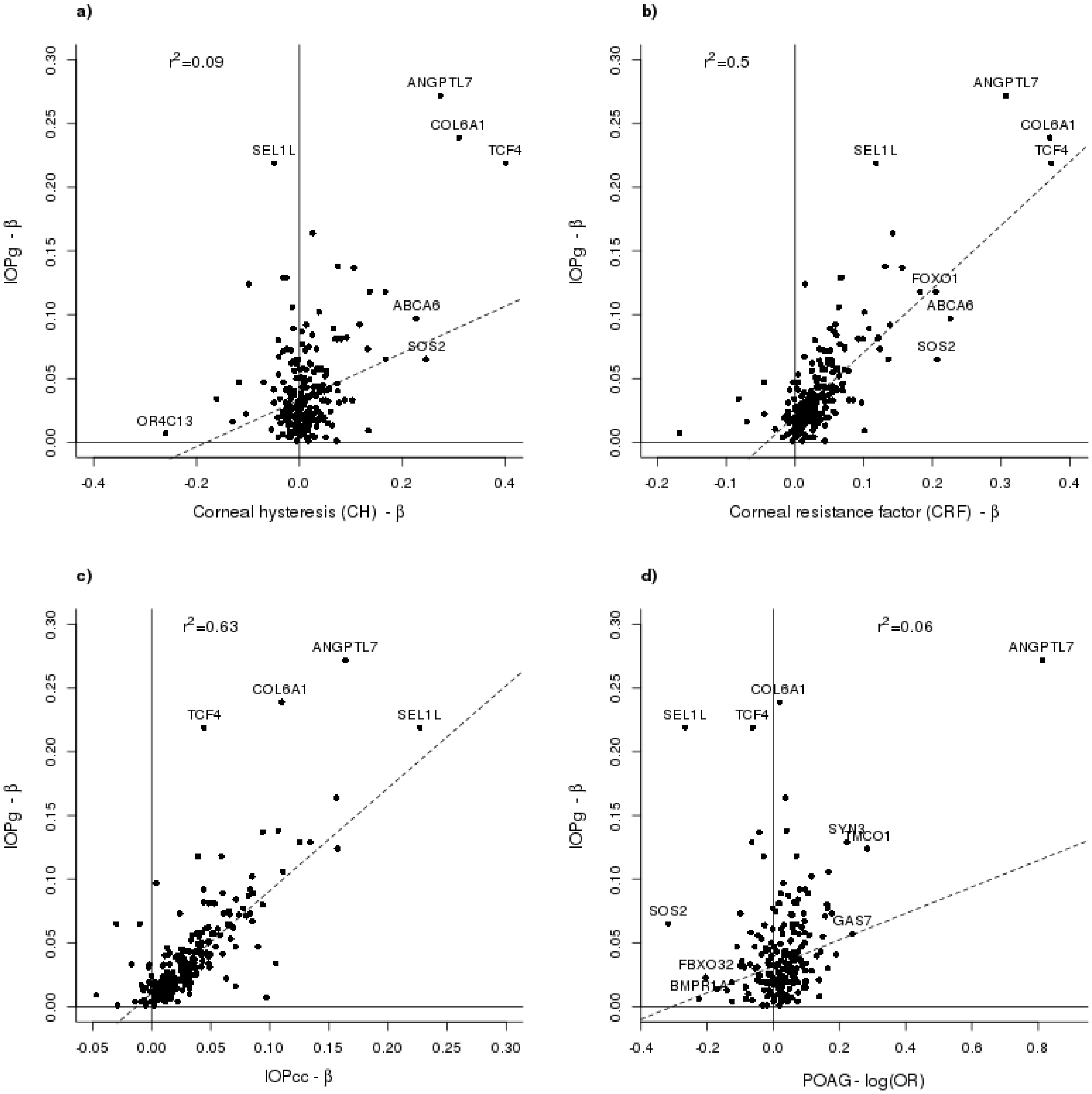

Supplementary Figure 15. Reported intraocular pressure variants and their effect on Goldmann correlated intraocular pressure (IOPg) vs their effect on hysteresis (CH), corneal resistance factor (CRF), corneal compensated intraocular pressure (IOPcc) and primary open angle glaucoma (POAG). Scatter plot showing the effect of 175 variants reported to associate with IOP[4] on IOPg against their effect on a) CH, b) CRF, c) IOPcc and d) POAG in our data. Effects are given for the allele that increases IOPg in our data.

Supplementary Tables

Supplementary Table 1. Correlation matrix for corneal measures. Pierson's correlation coefficients (r) and corresponding *P*-values are shown for all corneal traits obtained from the specular microscopy equipment and the ocular response analyzer. Correlation coefficients were computed after standardization and adjusting the measurements for age and sex. Correlation coefficients are shown in bold for significant correlations after adjusting for multiple testing with a false discovery rate procedure.

	CD		CV		CT		HEX		CH		IOPg		IOPcc	
	r	*P*-value	r	*P*-value	r	*P*-value	r	*P*-value	r	*P*-value	r	*P*-value	r	*P*-value
CV	**-0.33**	1.5E-155												
CT	**0.10**	8.0E-14	**0.04**	5.3E-03										
HEX	**0.15**	1.3E-30	**-0.65**	<1E-300	**0.04**	2.3E-03								
CH	**-0.03**	1.0E-02	**0.08**	2.2E-09	**0.53**	<1E-300	-0.01	6.6E-01						
IOPg	**-0.03**	9.6E-03	0.02	8.3E-02	**0.46**	<1E-300	**0.06**	2.2E-06	**0.14**	4.2E-26				
IOPcc	-0.01	3.0E-01	-0.01	3.3E-01	**0.23**	4.4E-73	**0.06**	1.3E-06	**-0.27**	3.9E-103	**0.91**	<1E-300		
CRF	**-0.04**	2.3E-03	**0.07**	5.5E-08	**0.63**	<1E-300	**0.03**	1.9E-02	**0.71**	<1E-300	**0.75**	<1E-300	**0.44**	2.3E-289

Supplementary Table 2. Significance thresholds for each variant category. The thresholds were estimated from the Icelandic data as previously described[5].

Category	Variants in category	Broad DHS	Significance threshold
High impact	Splice donor variant, splice acceptor variant, stop gained, frameshift variant, stop lost, initiator codon variant.	-	2.5×10^{-7}
Moderate impact	Inframe indels, missense, splice region variants, stop retained variants.	-	5.0×10^{-8}
Low impact	Synonymous variants, 5' UTR variant, 3' UTR variant, up- and downstream variants, coding sequence variants.	-	4.5×10^{-9}
Other	Intronic, intergenic variants.	Yes	2.3×10^{-9}
		No	7.5×10^{-10}

DHS = Dnase I hypersensitivity sites

Supplementary Table 3. Meta-analysis. Number of cases and controls for each phenotype we tested in a meta-analysis of GWAS results from Iceland and the UK Biobank.

		Iceland		UK Biobank		Total	
**Categorical Trait**	**ICD 10 code**	**N cases**	**N controls**	**N cases**	**N controls**	**N cases**	**N controls**
Glaucoma	H40	4004	237214	4428	404139	8432	641353
Primary open angle glaucoma (POAG)	H40.1	1261	303388	1035	402549	2296	705937
Primary angle closure glaucoma (PACG)	H40.2	78	229149	699	407868	777	637017
Disorders of cornea	H18	613	328012	623	407944	1236	663218
Corneal degenration	H18.4	81	287394	118	396627	199	684021
Hereditary corneal dystrophies	H18.5	119	304665	211	378987	330	683652
Keratoconus	H18.6	46	251017	81	408486	127	659503
**Quantitative Trait**		**N**		**N**		**N**	
Height		82556		407825		490381	

Supplementary Table 4. The association of corneal structure variants on all corneal measures and ocular diseases. Effects are given for the minor allele. Effects and *P*-values are shown in bold for genome-wide significant associations.

	ANAPC1		TCF4		17p12		11q24.1		8p23.1		ZNF469		WNT10A		COL5A1		ADAMTS17		GLT8D2	
	P-value	Effect	P-value	Effect	P-value	Effect	P-value	Effect	P-value	Effect	P-value	Effect	P-value	Effect	P-value	Effect	P-value	Effect	P-value	Effect
Cell density	**1.8E-314**	**-0.77**	**1.6E-19**	**-0.39**	9.3E-02	-0.03	1.0E-02	-0.07	6.5E-01	-0.01	2.2E-02	-0.05	9.4E-02	-0.10	4.7E-01	-0.02	8.3E-01	-0.01	4.5E-01	-0.04
CV	2.8E-28	0.23	4.1E-03	0.12	**6.9E-13**	**0.14**	**3.3E-10**	**0.16**	**7.6E-12**	**0.15**	8.5E-01	0.00	2.2E-01	-0.07	6.0E-01	-0.01	9.8E-01	0.00	7.6E-01	0.01
CCT	1.2E-01	-0.03	5.4E-01	-0.03	6.7E-02	-0.04	1.6E-02	-0.06	2.6E-03	0.07	**1.9E-14**	**-0.15**	**4.5E-11**	**-0.39**	**6.1E-11**	**-0.14**	**1.3E-10**	**0.18**	**3.9E-10**	**0.30**
HEX	2.8E-13	-0.16	5.9E-18	-0.37	5.4E-04	-0.07	5.4E-04	-0.09	1.5E-02	-0.05	3.2E-01	0.02	2.8E-02	0.13	6.4E-01	-0.01	1.2E-01	-0.04	9.3E-01	0.00
CH	2.6E-19	0.19	3.1E-12	-0.29	3.9E-02	0.04	1.8E-01	-0.03	1.1E-01	0.04	2.4E-07	-0.10	3.5E-04	-0.21	1.7E-01	-0.03	1.1E-04	0.11	6.4E-01	-0.02
IOPg	3.6E-01	0.02	1.7E-05	-0.18	9.4E-01	0.00	7.6E-02	-0.04	4.0E-01	-0.02	5.1E-03	-0.06	4.1E-01	0.05	4.9E-01	-0.02	5.8E-07	0.14	2.7E-01	0.05
IOPcc	4.5E-03	-0.06	1.9E-01	-0.05	4.5E-01	-0.02	2.1E-01	-0.03	1.2E-01	-0.03	4.4E-01	-0.02	1.8E-02	0.14	7.8E-01	-0.01	5.8E-04	0.09	2.1E-01	0.06
CRF	9.9E-11	0.14	7.9E-13	-0.30	1.8E-01	0.03	1.2E-01	-0.04	6.2E-01	0.01	3.2E-07	-0.10	7.2E-02	-0.11	1.5E-01	-0.03	1.4E-08	0.16	9.7E-01	0.00
Disorders of cornea (H18)	4.3E-01	0.95	1.4E-02	1.78*	8.9E-01	0.99	4.7E-02	1.13	3.2E-01	0.94	8.0E-03	1.35	1.6E-01	1.55	3.1E-01	0.94	1.7E-01	0.89	7.4E-01	0.94
Corneal degenration (H18.4)	8.6E-01	0.98	**9.9E-09**	3.98*	5.5E-01	1.07	5.9E-03	1.39	9.2E-01	0.99	6.8E-02	0.97	9.3E-01	1.01	4.5E-01	1.09	8.2E-01	0.96	6.4E-01	1.15
Hereditary corneal dystrophies (H18.5)	3.0E-02	0.81	**3.3E-31**	7.77*	2.0E-01	0.90	5.6E-01	0.94	2.1E-01	0.89	3.7E-01	1.08	6.2E-01	1.13	5.1E-01	0.94	1.4E-01	0.82	8.2E-01	0.94
Keratoconus (H18.6)	5.1E-01	0.88	5.1E-01	1.35*	8.8E-01	0.98	3.3E-01	1.16	1.4E-01	0.80	2.7E-02	0.73	4.3E-01	1.35	6.2E-03	1.47	1.3E-01	0.71	6.6E-01	0.82
Glaucoma (H40)	4.8E-02	1.04	1.5E-01	0.92*	5.8E-01	0.99	4.2E-01	1.02	2.9E-01	1.02	3.4E-01	1.05	4.0E-01	1.14	7.9E-01	0.99	8.3E-01	1.01	6.7E-01	1.02
POAG (H40.1)	2.4E-01	0.96	5.1E-01	0.94*	5.4E-01	0.98	3.4E-01	1.04	3.4E-01	1.03	8.7E-02	0.95	6.2E-01	1.05	6.9E-01	1.02	3.7E-01	0.96	9.2E-01	1.01
PACG (H40.2)	1.9E-01	0.92	2.4E-01	0.61*	6.9E-01	0.98	7.9E-01	1.02	7.1E-01	0.98	7.5E-01	0.98	9.9E-01	1.00	7.4E-01	0.98	6.3E-01	1.04	7.1E-01	0.93

*Effect and P-value was estimated with Icelandic data only.

Supplementary Table 5. Association of novel CCT variants with CCT in the Reykjavik Eye Study (N=1,459). Effects are given for the minor allele.

Chr	Pos	rs-name	Allele (min/maj)	MAF (%)	Gene	Coding effect	P-value	Effect
15	100152748	rs72755233	(A/G)	13.8	ADAMTS17	missense	1.21E-02	0.145
12	104015054	rs117801489	(C/T)	4.3	GLT8D2	missense	7.89E-03	0.258

Supplementary Table 6. Mutations in *CHST6* known to cause macular corneal dystrophy (MCD) under the recessive model. a) The standardized values of corneal measures for two homozygote carriers of the frameshift mutation p.Val6MetfsTer106 in *CHST6*. b) The association of MCD variants in *CHST6* with corneal measures under the additive model.

a)	Subject	Sex	Age	CD (SD)	HEX (SD)	CV (SD)	CCT (SD)	CH (SD)	IOPcc (SD)	IOPg (SD)	CRF (SD)
	1	Male	44	-3.38	-0.81	-1.12	0.76	-0.78	1.88	1.72	1.78
	2	Male	51	-2.65	-3.18	2.97	2.31	-1.54	0.18	-0.51	-2.32

b)		CCT		CD		CV		HEX	
**Variant**	**MAF (%)**	**P-value**	**Effect**	**P-value**	**Effect**	**P-value**	**Effect**	**P-value**	**Effect**
p.Ala128Val	0.662	0.42	-0.10	0.54	-0.07	0.05	0.23	0.21	-0.15
p.Val6MetfsTer106	0.072	0.29	0.34	0.01	-0.83	0.08	0.54	0.07	-0.56

CD = cell density; HEX = percentage of hexagonal cells; CV = coefficient of cell size variation; CCT = central corneal thickness; CH= corneal hysteresis; IOPg = Goldmann correlated intraocular pressure; IOPcc = corneal compensated intraocular pressure; CRF = corneal resistance factor.

Supplementary Table 7. Correlation between effect sizes. For known primary open angle glaucoma (POAG), central corneal thickness (CCT) and intraocular pressure (IOP) variants, the table shows the correlations between their effects of POAG, CCT and IOP, respectively, and their effect on different corneal measures. *P*-values are shown in bold for significant correlations after controlling the false discovery rate at 0.05 using Benjamin-Hochberg procedure.

	POAG variants			CCT variants			IOP variants		
	Effect	P-value	R^2	Effect	P-value	R^2	Effect	P-value	R^2
POAG	-	-	-	-0.16	3.3E-01	0.02	0.10	**2.4E-04**	0.06
CD	-0.32	0.49	0.03	0.35	1.6E-01	0.04	-0.16	**3.4E-03**	0.04
CCT	-0.17	0.58	0.02	-	-	-	0.14	**2.6E-03**	0.04
CV	-0.65	0.27	0.09	0.11	5.4E-01	0.01	-0.04	5.3E-01	0.00
HEX	0.35	0.57	0.02	-0.09	6.4E-01	0.00	0.05	3.7E-01	0.00
CH	-0.20	0.51	0.03	0.50	**1.4E-03**	0.19	0.18	**1.5E-05**	0.09
CRF	0.18	0.63	0.02	0.55	**3.6E-04**	0.24	0.50	**4.1E-33**	0.50
IOPcc	0.94	0.03	0.31	0.34	1.5E-01	0.04	0.81	**8.4E-48**	0.63
IOPg	0.83	0.06	0.22	0.65	**1.6E-03**	0.19	-	-	-

POAG = primary open angle glaucoma; CD = cell density; CCT = central corneal thickness; CV = coefficient of cell size variation; HEX = percentage of hexagonal cells; CH= corneal hysteresis; CRF = corneal resistance factor; IOPcc = corneal compensated intraocular pressure; IOPg = Goldmann correlated intraocular pressure.

Supplementary Table 8. Fraction of variance explained. Fraction of variance explained was calculated for all variants in GWAS catalog[6] that associated with a quantitative trait where corresponding measurements were available in deCODE data, using the Icelandic effects and MAF. The table shows the variant explaining highest fraction of variance for the top 10 traits.

Chr	Pos	Gene	Quantitative trait	Fraction of variance explained
6	160589086	LPA	Lp (a) levels	19.5%
2	233763993	UGT1A1	Bilirubin levels	6.6%
3	133758857	TF	Iron status biomarkers (transferrin levels)	4.1%
5	177415473	F12	Activated partial thromboplastin time	2.6%
19	48703160	FUT2	Vitamin B12 levels	2.5%
2	43847292	ABCG8	Sitosterol	2.5%
19	44909976	APOC1	LDL cholesterol	2.4%
16	56961324	CETP	HDL cholesterol	2.2%
4	9920543	SLC2A9	Uric acid levels	1.9%
12	121779004	TMEM120B	Mean platelet volumn	1.9%

Supplementary references

1. Wood, A. R. *et al.* Defining the role of common variation in the genomic and biological architecture of adult human height. *Nat. Genet.* **46,** 1173–1186 (2014).
2. Iglesias, A. I. *et al.* Cross-ancestry genome-wide association analysis of corneal thickness strengthens link between complex and Mendelian eye diseases. *Nat. Commun.* **9,** 1–11 (2018).
3. Lu, Y. *et al.* Genome-wide association analyses identify multiple loci associated with central corneal thickness and keratoconus. *Nat. Genet.* **45,** 155–163 (2013).
4. Gao, X. R., Huang, H., Nannini, D. R., Fan, F. & Kim, H. Genome-Wide Association Analyses Identify New Loci Influencing Intraocular Pressure. *Hum. Mol. Genet.* **0,** 1–9 (2018).
5. Sveinbjornsson, G. *et al.* Weighting sequence variants based on their annotation increases power of whole-genome association studies. *Nat. Genet.* **48,** 314–317 (2016).
6. MacArthur, J. *et al.* The new NHGRI-EBI Catalog of published genome-wide association studies (GWAS Catalog). *Nucleic Acids Res.* **45,** D896–D901 (2017).
