# Supplementary material for: Sequence variation at ANAPC1 accounts for 24% of the variability in corneal endothelial cell density
Source: Nat Commun. 2019 Mar 20;10:1284. doi: 10.1038/s41467-019-09304-9 (PMC6427039; doi:10.1038/s41467-019-09304-9)
Supplement: Supplementary file 3 — Description of Additional Supplementary Files [file 41467_2019_9304_MOESM3_ESM.pdf]

Description of Additional Supplementary Files

Supplementary Data 1

Tissue expression of genes at the associating loci according to the Ocular Tissue Database. In the Ocular Tissue Database (OTDB), the gene expression is indicated as Probe Logarithmic Intensity (PLIER) normalized value.

Supplementary Data 2

Previously reported POAG variants and their effect on corneal measures. POAG replication *P*-values are shown in bold for nominal significant associations ($P<0.05$).

Supplementary Data 3

Previously reported CCT variants and their effect on corneal measures. CCT replication *P*-values are shown in bold for nominal significant associations ($P<0.05$).

Supplementary Data 4

Previously reported IOP variants and their effect on corneal measures. IOP replication *P*-values are shown in bold for nominal significant associations ($P<0.05$). The variants and their reported effects were obtained from Gao, X.R. et al. (2018).

Supplementary Data 5

GWAS summary statistics for cell density, CV, HEX and CCT are provided for all associations with *P*-value below $1 \cdot 10^{-6}$.
